# Scaffolds as Structural Tools for Bone-Targeted Drug Delivery

**DOI:** 10.3390/pharmaceutics10030122

**Published:** 2018-08-08

**Authors:** Riccardo Ferracini, Isabel Martínez Herreros, Antonio Russo, Tommaso Casalini, Filippo Rossi, Giuseppe Perale

**Affiliations:** 1 Department of Surgical Sciences, Orthopaedic Clinic-IRCCS A.O.U. San Martino, 16132 Genova, Italy; riccardoferraciniweb@gmail.com (R.F.); 4584108@studenti.unige.it (I.M.H.); russo.antonio.92@gmail.com (A.R.); 2 Department of Chemistry and Applied Biosciences, Institute for Chemical and Bioengineering, ETH Zurich, Vladimir-Prelog-Weg 1, 8093 Zurich, Switzerland; tommaso.casalini@chem.ethz.ch; 3 Biomaterials Laboratory, Institute for Mechanical Engineering and Materials Technology, University of Applied Sciences and Arts of Southern Switzerland, Via Cantonale 2C, Galleria, 26928 Manno, Switzerland; 4 Department of Chemistry, Materials and Chemical Engineering “Giulio Natta”, Politecnico di Milano, via Mancinelli 7, 20131 Milano, Italy; filippo.rossi@polimi.it; 5 Industrie Biomediche Insubri SA, Via Cantonale 67, 6805 Mezzovico-Vira, Switzerland

**Keywords:** biomaterials, bone, polymer, scaffold, stem cell

## Abstract

Although bone has a high potential to regenerate itself after damage and injury, the efficacious repair of large bone defects resulting from resection, trauma or non-union fractures still requires the implantation of bone grafts. Materials science, in conjunction with biotechnology, can satisfy these needs by developing artificial bones, synthetic substitutes and organ implants. In particular, recent advances in materials science have provided several innovations, underlying the increasing importance of biomaterials in this field. To address the increasing need for improved bone substitutes, tissue engineering seeks to create synthetic, three-dimensional scaffolds made from organic or inorganic materials, incorporating drugs and growth factors, to induce new bone tissue formation. This review emphasizes recent progress in materials science that allows reliable scaffolds to be synthesized for targeted drug delivery in bone regeneration, also with respect to past directions no longer considered promising. A general overview concerning modeling approaches suitable for the discussed systems is also provided.

## 1. Introduction

Bone diseases are among the most common conditions threatening human health worldwide, becoming a major socioeconomic and global health care problem [1,2,3]. Bone tissue has the remarkable ability to regenerate itself after an injury, through complex and strictly regulated biological processes. Although, in some circumstances, such as extensive bone resections due to bone cancer, osteoporosis, osteomalacia, osteomyelitis, avascular necrosis, and atrophic non-union, bone regeneration could be impaired [4]. Thus, in these cases bone grafts are often needed in order to restore the normal function and native biomechanical characteristics of the affected bone. Autologous grafts are the clinical gold standard to improve bone regeneration, since they are osteoinductive, osteoconductive and totally histocompatible, reducing the risk of immunogenic reactions and disease transmission. However, despite these properties, autografts still show some limitations due to the little amount of tissue available for grafting, donor site morbidity and the need for additional surgery, which may lead to an implant failure.

An alternative approach is represented by allogenic bone grafts, but possible immune reactions and disease transmission are still important issues to solve [5,6,7,8].

**Figure 1:**
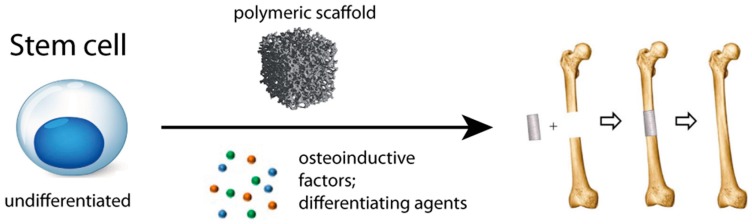
Tissue engineering approach to bone repair: undifferentiated stem cells were seeded within polymeric scaffolds, together with differentiating agents (e.g., dexamethasone) and osteoinductive agents and then implanted in vivo.

Bone tissue engineering (BTE) is a valid emerging approach for bone regeneration, aiming to overcome these problems (Figure 1). In contrast with most traditional approaches, BTE contemplate the use of porous structures, called scaffold, which serve as a template to guide cell attachment, differentiation, proliferation and tissue regeneration [9]. An ideal scaffold should have some particular properties like: a good biocompatibility, meaning that the scaffold should be able to interact with the cellular component of bone, without leading any toxic or immunological response; a suitable porosity, that mimics the natural structure of bone tissue, which permit vascular ingrowth and cellular transportation; adequate mechanical properties; and biodegradability, in fact an ideal scaffold is expected to degrade over time in vivo, preferably at a predictable absorption rate [10,11,12,13]. So far, many materials that possess some of these properties have been studied for applications in BTE, but the development of a scaffold which can fulfill all of the above requirements is still challenging. More recently, much interest has been addressed in exploiting scaffolds for delivering targeted therapies to bone, in order to supply molecules like growth factors, which are fundamental to assist and enhance bone regeneration, or drugs and small molecules, which can locally treat bone disorders. Moreover, the intense research in integrating BTE with modern nanotechnologies has been demonstrating to be a valid strategy in delivering therapeutic molecules to bone tissue [14]. An enormous literature is available on the topic, demonstrating the critical and promising role of these novel approaches to treat skeletal disorders.

In this review, we summarize the most-studied materials for designing scaffolds in BTE, their role as targeted drug carriers and pharmaceutics agents proposed for treating bone regeneration defects. The eventual implications of this approach in the clinic are also assessed.

## 2. Biomaterials for Bone Scaffolds

### 2.1. Organic Scaffolds

#### 2.1.1. Synthetic Polymers 

Synthetic polymers have demonstrated being promising biomaterials for bone tissue engineering, due to their biomechanical and biodegradability properties. Moreover, they provide a better controllability in terms of porosity, physiochemical structure and immunologic adverse effects when compared to other types of scaffolds [15,16,17]. The most studied synthetic polymers in bone tissue regeneration are aliphatic polyesters like poly(lactic-acid) (PLA), poly(caprolactone) (PCL), and poly(glycolic-acid) (PGA) and derivatives. 

Other synthetic polymers include poly(methyl methacrylate), poly(e-caprolactone), poly hydroxyl butyrate, polyethylene, polypropylene, polyurethane. These polymers are degraded by hydrolysis in vivo and have the advantage of being easily tailored in different shapes, according to mechanical demands in the particular bone treated [18,19,20,21]. However, synthetic polymers still cause some concern about the timing of absorption, which can alter their mechanical strength in vivo. 

Some polymers, such as poly(propylene fumarate) (PPF) demonstrated great resistance to compressive stress and a controlled biodegradability; however, their degradation leads to the release of acid compounds that could constitute an adverse issue on native bone health [21]. 

#### 2.1.2. Natural Polymers

The rationale of studying the role of natural polymers in bone tissue regeneration lies in their similarity to native extracellular matrix (ECM) and, according to their chemical composition, they can be divided in proteins (collagen, gelatin, fibrinogen, elastin) and polysaccharides (glycosaminoglycans, cellulose, amylose) [20,22]. Their resemblance to the native ECM makes them scaffolds with high osteoinductive properties and biocompatibility. Several strategies have been proposed for fabricating natural polymeric scaffolds: they can be derived by cells, which are inducted to produce ECM, or directly obtained from decellularized bone tissue [23]. ECM-based scaffolds are generally highly biocompatible and display poor or no risk of host immune reaction; however, the need of an additional surgery to sample grafts, with consequent loco-regional morbidity and limited availability of tissue have not been of negligible limitations to this approach. Allografts, xenografts and demineralized bone matrices in contrast show no concerns about availability of biomaterials, and have high osteoinductive and osteoblast stimulation properties; however, possible host immune reaction and risk of disease transmission still are concerning. Natural polymers scaffolds demonstrated to provide mesenchymal stem cells differentiation to osteoblast and have shown a high biocompatibility. However, mechanical properties and biodegradability are still less controllable in naturally derived biomaterials compared to synthetic polymers [24]. Chitosan (CS) is a deacetylated derivative of chitin and is used as a vehicle for drug delivery, being able to enhance the absorption of hydrophobic macromolecular drugs. CS nanoparticles (CSNPs) are used to reduce the toxic effects of a drug and extending its activity by holding the therapeutic agent in closer proximity to the site of action, due to its muco-adhesive cationic nature [25]. CSNPs are usually modified in order to enhance cell internalization efficiency. For instance, CSnP can be altered with sulfate groups to create a polysaccharide comparable in structure to heparin, which can link favorably to the basic amino-acid stretches of BMP-2. By improving the sustained release, this interaction can enhance the bioactivity of BMP-2 for the healing of the bone [26].

#### 2.1.3. Lipid Nanoparticles

Lipid nanoparticles (LNPs), have great potential in comparison with inorganic nanoparticles, due to their biocompatibility and low toxicity. LNPs are uniform nano-carriers including solid- and liquid-state lipids, that can be organized into a core-shell or homogeneous particle structure [27]. They include lipid drug conjugate (LDC) carriers, nanostructured lipid carriers (NLCs), lipid nanocapsules carriers (LNCs) and solid lipid nanoparticle (SLN) carriers [28].

#### 2.1.4. Purified Bone Allografts

Different industrial processes exist to purify allogenic bone, i.e., from foreign donors, providing safe and cleaned blocks for bone allografting, many of which are also marketed even if possible host immune reaction and risk of diseases transmission still are concerning and are resulting in continuously more stringent regulatory restrictions. Moreover, although allografts could potentially be true biological scaffolds, cleaning processes often make use of chemical reagents that are aggressive for the bone matrix and, hence, result in poorly performing materials both mechanically and biologically [29]. 

However, being still used, these types of graft are investigated also for local drug delivery purposes, particularly antibiotics, although with contrasting results: loading of allografts with antibiotics may not only protect grafts from bacterial adhesion but should be above the minimum biofilm eradication concentration for a prolonged period of time to be efficient [30].

### 2.2. Inorganic Scaffolds 

#### 2.2.1. Metallic Scaffolds

##### Mesoporous Silica Nanoparticles 

Mesoporous silica nanoparticles (MSNs) have a number of features that make them specifically interesting as a targeted delivery vector. MSNs are able to load diverse cargos because of the pore size and morphology of MSNs. Surface-modified MSNs can release their cargo under control. Multifunctional (magnetic and luminescent) MSNs have the possibility of bio-imaging and drug delivery at the same time. The alteration of MSNs, with appropriate functional groups (such as –NH_2_, –Cl, –SH, or –CN) or polymers, directly influence the drug release rate by rising the drug diffusion resistance. MSN-based multifunctional drug delivery systems are able to deliver antitumor drugs in a targeted fashion and release them on demand to increment their cellular uptake without any premature release prior to reaching the target site [31]. They can accelerate bone formation by upregulating osteoblast activity and decreasing bone resorption by downregulating osteoclast activity; for this reason they are still a great option for treating osteoporosis [27]. 

##### Gold Nanoparticles 

Gold nanoparticles (GNPs) are adequate for controlled drug delivery, cancer treatment, biomedical imaging, diagnosis (due to their excellent compatibility with the human organism), low toxicity, small dimensions, and the capacity of being encased with a variety of therapeutic agents [32]. GNPs can downregulate the formation of osteoclasts by inhibiting the function of the osteoclastogenesis promoter, RANK ligand (RANKL), and by reducing reactive oxygen species (ROS) levels [33]. GNPs can be used with drugs or other molecules for drug delivery. To induce osteogenic differentiation, they can provide mechanical stress on the membranes of mesenchymal stem cells (MSCs) to activate the mechanosensitive p38 mitogen-activated protein kinase (MAPK) pathway [34,35].

##### Nanodiamonds

Nanodiamonds (NDs) are octahedral, nanoscale carbon allotropes that are perfect intracellular carriers of bioactive compounds because of their properties such as: biocompatibility, reduced dimensions and high surface chemical interaction [27]. NDs have been suggested to have a positive role in osteoblast proliferation and differentiation but appears to have gained overall not much interest for long term translational studies [36].

#### 2.2.2. Ceramic Scaffolds

Bone tissue has the particular feature of combining biological components like osteocites, osteoblasts and osteoclast to an abundant inorganic extracellular matrix, being composed of almost 70% hydroxyapatite and 30% collagen in weight [37]. Calcium phosphate nanoparticles (CPNs) have received great attention for bone-related applications because of their superior biocompatibility, biodegradability, and similarity in structure to the inorganic composition of bone minerals [38].

Ceramic scaffolds derive from CaP and, due to their analogy in physicochemical composition to the inorganic component of bone, they have been extensively studied as substrates for BTE [39]. The most investigated CaP scaffolds are hydroxyapatite (HA), beta-tricalcium phosphate (b-TCP), and a combination of HA and b-TCP, called bifasic calcium phosphate (BCF) [40]. Ceramic scaffolds demonstrated of being able to integrate in natural bone tissue and stimulate osteoblast differentiation, osteoblast growth and inorganic matrix deposition. However, clinical application of CaP scaffolds is limited by their fragility, irregular absorption rate and overall poor clinical outcomes. Thus, new bone tissue formed in a ceramic scaffold cannot sustain mechanical loading as well as natural bone. More recently, it has been proved that doping CaP scaffold with various compounds could improve mechanical resistance, biocompatibility and absorption rate. For instance, Fielding et al. demonstrated that the addiction of SiO_2_ and ZnO to b-TCP increase compressive strength to 2.5-fold and cell viability to 92% [41], however, clinical evidence is still lacking. Further new approaches to the problem of brittleness of ceramic scaffolds include composite materials, in which CaP are mixed with organic polymers. Composite scaffolds also demonstrated a promising role in drug delivery, thanks to their porosity and cell adhesion ability [42].

#### 2.2.3. Composite Xenohybrid Scaffolds

It is a generally accepted paradigm that bone substitutes should resemble as closely as possible naturally occurring human cancellous bone and a very commonly used source of bone matrices are animal-derived bones, where mostly used in clinical practice are bovine xenografts, distantly followed by equine and porcine. It is acknowledged that bovine-derived cancellous bone grafts are the closest xenograft to human bone to be regenerated, second only to autografts [43,44] and are products used in clinical practice where bone regeneration is needed in reconstructive surgeries [45,46]. However, necessary cleaning and sterilization processes of starting raw materials of animal origin result in a decay of both mechanical and biological performances. Indeed, similarly to what is seen for synthetic biomaterials, the application of composite technologies to bovine xenografts is becoming an interesting trend, not only in research [47,48] but also in industrial and clinical practices [49,50]: the adding of resorbable polymeric components improves mechanical and biological performances [51] and can also be used to locally carry active molecules, or drugs to be delivered locally, to increase cell colonization, promote osteoinduction and finally promote osteogenesis [52,53], hence, representing an increasingly interesting family of biomaterials.

## 3. New Pharmaceutic Agents in Bone Targeted Therapies

Several drug therapies have been studied for their potential in enhancing bone tissue healing. Historically, growth factors are the agents that have attracted greater attention, since they are fundamental for bone regeneration. Bone morphogenetic protein-2 (BMP-2) and BMP-7 are the most extensively investigated growth factors due to their ability to promote mesenchymal stems cell (MSC) differentiation to osteoblasts, although often with debatable or contradictory results [54]. Parathyroid hormone (PTH), which has already been approved for the treatment of osteoporosis, could be also a therapy for bone healing defects [55,56]. Biphosphonates have demonstrated being drugs for targeting bone and are among the most studied molecules in BTE. Several studies proved their efficacy in carrying antibiotics or chemotherapy agents to bone, thanks to their affinity to hydroxyapatite [57]. Antibodies have also been suggested as a model to target bone in diverse conditions. In addition, novel approaches including small molecules like statins, steroids, prostaglandins agonists, and Wnt/beta-catenin agonists have been explored, as they are relatively stable, affordable and display little immunogenicity [58,59,60]. 

An alternative strategy is represented by the delivery of nucleotides, like siRNA and miRNA, which can be used to genetically rearrange cells acting at the site of healing, thus improving regeneration of bone tissue [61,62].

### 3.1. Growth Factors and PTH

Growth factors are signaling proteins able to stimulate the proliferation and differentiation of target cells, through a complex control of the cell cycle and gene expression. Several growth factors are involved in bone maturation and normal healing processes, explaining the vast interest they received as therapeutic agents in bone healing defects [54,63,64]. Systems for the delivery of recombinant human BMP-2(rhBMP-2) and BMP-7 (rhBMP-7) have already been approved by the FDA for clinical uses [65,66]. Jones et al. in 2006 demonstrated that a combination of allograft and rhBMP2-INFUSE™ significantly improved bone healing in diaphyseal tibia fractures, when compared to autograft approaches [67]. However, the administration of rhBMPs is still not approved in pediatric patients, in pregnant women and in patients with tumors, due to the concerning side-effects they can display [68,69]. More recently, several nanostructures have been developed for the delivery of BMP-2 and BMP-7 to bone, in order to overcome uncontrolled diffusion of the growth factors and consequent adverse effects. Organic polymers and hydrogels are the most studied materials in the field of BMPs controlled delivery to bone, since their chemical properties can be modulated incorporating functional groups that make polymers susceptible to degradation at particular pH and temperature levels. For instance, a pH-responsive and thermosensitive hydrogel, designed by adding sulfamethazine oligomer (SMO) to copolymers made of PCLA and PEG units, showed a functional release of BMP-2, leading to increased ALP activity and mineralization after subcutaneous administration [70]. PTH is a peptidic hormone the action of which is crucial in regulating calcium and phosphorus omeostasis. Systemic subcutaneous injections of PTH, and its derivative human recombinant PTH (hrPTH 1-34), are FDA-approved for the treatment of osteoporosis, and have been used as effective off-label treatments of fracture healing [56]. Furthermore, experimental studies conducted in animal models of multiple fractures demonstrated that PTH enhanced callus mineralization and bone remodeling [55,71]. Local administration of PTH to sites of fractures is now being explored. An arginine-glycine-aspartic acid (RGD)-modified PEG hydrogel bound to hrPTH 1-34 was demonstrated to induce bone formation comparable to autologous bone graft implantation, in a canine model of dental implants [72]. However, some concerns are emerging about the timing of degradation of PTH during storage in such carriers and further studies on the role of polymers for PTH delivery are needed for translation to clinical practice [73]. 

### 3.2. RNA Interference (RNAi)

RNA interference is a phenomenon first described by Fire and coworkers in which short sequences of non-coding RNA, such as siRNA and miRNA, are able to alter gene expression in target cells [71]. The mechanism by which siRNA acts is complex: long molecules of double strand RNA (dsRNA) are cleaved by an endo-ribonuclease called Dicer into siRNA molecules, which are then free to be enclosed into a protein complex named RNA-Induced Silencing Complex (RISC). Once the RISC is formed, it binds to target complementary mRNA which is then degraded, suppressing its translation into amino acids. The mechanism of miRNA is even more complex, since miRNA derives from long coding-dsRNA primary transcript, called pri-miRNA, that then undergoes extensive post-transcriptional modifications which leads to the formation of pre-miRNA. The conversion of pri-miRNA into pre-miRNA is mainly due to the action of an RNase III named Drosha and an RNA-binding protein, DGCR8. The pre-miRNA sequence is then cleaved by Dicer, leading to the formation of mature miRNA, which can be integrated in the RISC, that seems to be the final common pathway of RNAi phenomenon. The rationale of studying RNAi in the context of BTE is to manipulate cells involved in bone healing, in order to silence the expression of biological products that contrast new bone tissue deposition. 

For example, administration of siRNA against DKK-1 or guanidine nucleotide binding protein alpha stimulating activity polypeptide 1(GNAS1) showed an improved osteogenic differentiation of MSCs, suggesting that the application of RNAi technologies could be promising in BTE [74,75]. Nevertheless, clinical translation of RNAi is still challenging, since these short sequences of RNA are susceptible to nuclease action, and the absorption by targeted cells is hampered by cell and endosomal membrane impermeability [76].

More recently, nanotechnology has been exploited for RNAi delivery, aiming to overcome problems related to degradation by nuclease and cell membrane permeability. LNPs are the most studied nano-carriers both in vitro and in vivo, but some limitations derive from high-dose toxicity and adverse immune reaction [77,78]. Polymers such as PEI, poly-l-lysine, poly-l-arginine, chitosan, and derivatives have also been explored for the development of structures for siRNA delivery [79]. In particular, PEI showed favorable interactions with target cell membranes and high cell uptake. Its conjugation into photo-crosslinked dextran hydrogels through a biodegradable ester linker resulted in a sustained delivery of siRNA [80,81]. PLGA has also been employed for siRNA delivery, offering the advantages of high stability and low toxicity, but weak electrostatic interactions between PLGA and siRNA result in a limited delivery potential [82]. Several strategies have been developed to overcome this problem demonstrating optimized results, such as loading the surface of PLGA with polymeric NPs. Polymeric NPs are particularly promising systems in the field of siRNA delivery. 

For instance, in 2009, Convertine et al. developed a diblock copolymer delivery system containing a block of poly(dimethylaminoethyl methacrylate)(pDMAEMA) and a pH-responsive block of DMAEMA, 2-propylacrylic acid (PAA), and butyl methacrylate (BMA), which efficiently protected siRNA form degradation, improved cell uptake, realized endosomal escape, achieving an optimal siRNA delivery to MSCs [83,84]. Furthermore, the same delivery system was combined with siRNA against WW domain-containing E3 ubiquitin protein ligase 1 (Wwp1), a suppressor of osteoblasts, and then enclosed into PEG-derived hydrogels; when inserted at the site of murine femur fractures, it showed improved bone deposition and increased biomechanical strength [59].

### 3.3. Small Molecules

The term “small molecule” includes a variety of drugs and active molecules, with different physicochemical properties, which have in common a molecular weight lower than 900 Daltons. Thanks to their low weight, small molecules are generally affordable, stable, osteoinductive at low doses, display lower risks in terms of immune responses [85] and, hence, represent a very promising research arena. Numerous small molecules with osteoinductive properties have been discovered in the last decades, and some of these are approved for clinical uses for other conditions [86,87]. Thus, another advantage of studying small molecules as a resource for BTE includes the large amount of available data about toxicity and pharmacokinetic in humans, permitting a prompt translation to orthopedic applications. For instance, statins, which are 3-hydroxy-3-methylglutaryl coenzyme A (HMG-CoA) inhibitors commonly used for the treatment of different types of dyslipidemia, exhibited osteogenic potential both in vitro and in vivo [88]. In a murine mandibular bone defect model, a gelatin sponge graft coupled with simvastatin showed an increased new bone formation, compared to the control group [89]. Similar results have been obtained in experimental studies assessing bone healing by radiographs and histology [90]. However, some concerns derive from an in vivo study that showed inflammatory responses and impaired bone healing when statins when administered at high doses [91,92]. Therefore, more studies about the role of statins are needed to better understand this non-negligible point. Another approach extensively explored is the use of Wnt/β-catenin pathway agonists. Wnt/beta-catenin is a signaling pathway which plays a key-role in all stages of bone healing, by committing MSCs to osteoblast lineage and stimulating bone matrix deposition [93,94]. Lithium, which is already approved by FDA for the treatment of bipolar disorder, is able to inhibit glycogen synthase kinase-3 (GSK-3); thus, the inhibition of GSK-3 results in an over-expression of intracellular beta-catenin, that can promote and enhance bone regeneration [95]. 

Others GSK-3 inhibitors have been studied in pre-clinical rat model-based experiments, such as 603281-31-8 and AZD2858, exhibiting enhanced osteoblast activity and bone healing [96,97]. Some delivery systems have been developed in order to reduce drug dispersion and to better target bone healing site, like conjugating AZD2858 with poly(styrene-*alt*-maleic anhydride)-*b*-poly(styrene) (PSMA-*b*-PS) [98]. Nonetheless, there are still some concerns about clinical use of GSK-3 inhibitors, due to possible tumorigenic adverse effects, and more studied are necessary [99].

## 4. Clinical Applications in Drug Delivery

### 4.1. Osteomyelitis and Other Orthopaedic Related Infections (ODRIs)

*Staphylococcus aureus* is considered the causative agent of osteomyelitis for almost 80% of all cases of human disease [100]. *S. Aureus* has high affinity to bone, is able to induce osteonecrosis and resorption of the bone matrix [101]. The standard treatment consists in radical surgical debridement of the infected tissues, obliteration of the dead space, adequate soft tissue coverage, and intravenous antimicrobial therapy for at least 4–6 weeks [102]. Local delivery of antibiotics combined to biodegradable polymer scaffold for bone regeneration was envisioned as a new therapeutic strategy to overcome the limitations of systemic treatment and effectively manage osteomyelitis [103]. Local antibiotic delivery presents many advantages: the antibiotic reaches much higher concentration at the site of action, while keeping systemic antibiotic concentration at a minimum level, and adverse side effects commonly occurring with high systemic doses of antibiotics can be avoided. Biofilms, representing an aggregate of microorganisms embedded in a self-produced matrix of extracellular polymers, are responsible for most of the Staphylococcal infection of bone and ODRIs [104]. Literature data demonstrate that the presence of biofilm-embedded bacteria is more resistant to systemic antibiotic use because the biofilm decreases the local concentration of antibiotics at the bacterial surface. The use of local antibiotic carriers increases concentration of antibiotics overcoming the biofilm induced resistance [105]. The gold standard treatment for osteomyelitis and ODRIs is the use of antibiotic-loaded carriers consisting on inert materials for example cements or beads of polymethylmethacrylate (PMMA) impregnated with antibiotics capable to deliver high levels of antibiotic at a local administration site [40]. PLGA was employed to compose a poly(ethylene glycol) monomethyl ether (mPEG) and PLGA copolymer as a sol-gel drug delivery system for treating osteomyelitis. It is shown to have diverse advantages in treating osteomyelitis, including simple preparation, 100% encapsulation rate, near-linear sustained release of drugs, injectable design and in situ gelling of the target tissue [106].

### 4.2. Cancer Bone Metastasis

The most prevalent solid tumors, such as breast, lung and prostate cancers, metastasize to the skeleton and induce either osteolytic or osteoblastic lesions. Both types are often followed by bone pain and increased bone fragility, producing extended suffering [107]. The skeleton is the most typical organ to be affected by metastatic cancer, and this is the site of the disease that generates the major morbidity rates [108]. Treatment includes surgical management and radiation therapy and is aimed at the removal of cancer cells from a specific site and the prevention or treatment of impending fractures. The systemic approaches, consisting of hormone therapy, chemotherapy, systemic radionuclides and bisphosphonate therapy, can suppress the development of the tumor, providing symptomatic relief and regression of bone disease [109]. Bone-targeted drug delivery systems are used to concentrate chemotherapeutic drugs in bone tissues, reducing the adverse effects of neoadjuvant chemotherapy and solving the problem of reaching the desired foci [110]. Bisphosphonates (BPs) represent the most relevant drug family for the treatment of cancer bone metastasis. By decreasing osteoclast induced bone turnover, BPs can maximize structural bone strength, treat or prevent osteoporosis, and treat Paget’s disease of bone.

A case of intense and debated research was the suggestion that BPs might have antitumor properties and can possibly be used to treat cancer bone metastases [111,112]. Because of their great affinity to bone, BPs are used as targeting molecules on NPs to deliver anticancer drugs. Specifically, NPs were used to load doxorubicin (DXR) and were evaluated for their antitumor effects in primary or metastatic bone tumors in an orthotopic mouse model of breast cancer bone metastases. Both free DXR and DXR-loaded NPs demonstrate an important dose-dependent growth inhibition of the breast cancer cells [107]. Another study shown that a direct conjugate PTX–PEG–ALN (poly ethylene glycol bearing paclitaxel and alendronate) NP exhibited an improved pharmacokinetic profile due to the notable increase in their half-life. PTX is a potent anticancer drug that can result in serious side effects. Alendronate is an aminobisphosphonate used to treat osteoporosis, bone metastases, and bone targeting. This conjugate was shown to have a good binding affinity to the bone in vitro [113]. Some studies propose that targeting bone tissue biomarkers could represent another strategy to stop cancer bone metastasis. Therefore, combining delivery systems of NPs, selective drugs, and gene therapy may represent a new strategy to create effective treatments and prevention for bone metastasis [114,115]. 

### 4.3. Osteosarcoma and Other Musculoskeletal Malignancies

Osteosarcoma is the most common primary bone malignancy, often presented in the first or second decade of life [116]. It is treated using a surgical, neo-adjuvant and adjuvant chemotherapeutic regimen [117]. Locally advanced and metastatic soft tissue sarcoma have been managed only through surgery, radiotherapy and chemotherapy. Despite the efforts, overall 5-year survival rate in patients with soft tissue sarcomas of all stages remains only 50–60% [114]. The local relapses and systemic diffusion of bone and soft tissue sarcomas depend on the partial efficacy of the chemotherapeutic agents used. Their systemic and organ toxicity greatly limits the maximum tolerated dose of anti-cancer drugs and, thus, restricts their therapeutic efficacy. New treatment methods are still required to improve survival in patients with osteo- and soft-tissue sarcomas, the molecular targeted therapy may have a potential to be a promising therapy [118]. NPs can extravase and accumulate inside the interstitial space. This causes an enhanced permeability. Moreover, lymphatic vessels are absent or ineffective in tumors, conducing inefficient drainage of the tumor tissue [119]. Many small anti-cancer drugs have been encapsulated in PLGA based nanoparticles and have been studied in vitro and in vivo to treat different cancers: doxorubicin (DXR), a highly potent anthracycline approved for use against a wide spectrum of tumors is compromised by toxicities, cardiomyopathies leading to congestive heart failures. PEGylated PLGA nanoparticles encapsulating DXR enhance DXR anti-tumor efficacy compared with the free drug [119].

### 4.4. Osteoarthritis

Osteoarthritis is a prevalent, disabling disease leading to joint symptoms, signs associated with loss of integrity of the articular cartilage, and changes in underlying bone and joint surface [120]. Existent therapies have no effect on reverting or slowing down the progression of the disease and pursue only pain alleviation [121]. NPs could be useful as a local delivery system for osteoarthritis drugs, such as anti-inflammatory chemokines or chondrocyte-stimulating peptides, and this could increase the drug retention time in local tissues or fluids. For example, cationic polymeric hydrogel was reported to increase the retention time of dextran, after ionically cross-linked with the NP in synovial fluid without influence on the feature of the fluid [122]. Similarly, copolymer NPs were also shown to increase the retention time of IL-1 receptor antagonist (IL-1Ra) and maintained cartilage structure and composition [123]. 

### 4.5. Osteonecrosis

Osteonecrosis leads to osteoarthritis, especially in the hip and knee joints, during the third to fifth decades of human life. 

It depends on a vascular crisis inducing a massive remodeling of the vascular tree in the bone and of the bone architecture leading to a temporary weakness often causing a collapse of the affected subchondral bone. Although a number of surgical procedures have been developed, no single used treatment method has so far provided a cure in our hands. New treatment methods are required, such as biologic regeneration of necrotic bone [124]. The treatment using stem cells from bone marrow have been recently introduced in the clinical practice [125]. Moreover, PDLLA and PLGA had been utilized as promising means to deliver bioactive molecules. The inclusion of PDGF and simvastatin into PDLLA–PLGA microspheres proved the improvement of cellular viability and showed a decrease of inflammation by either simvastatin or PDGF treatment. In the osseous defect, release of PDGF in the early phases promotes cell recruitment, imitating the early mitogenic stage in wound healing. Simvastatin released after day 7, facilitates osteogenic differentiation and maturation [126].

### 4.6. Pseudo Arthrosis and Delayed-Non Unions

The late literature regarding the expected complications of the fractures identified clinical parameters where pseudo arthrosis or delayed unions are more frequent [127]. Actual treatment of the complications is reserved after the onset of the pathological healing process. The treatment requires curettage of the area and infusion of bone marrow derived stem cells [128,129]. The use of bone scaffold is a novel treatment under investigation [130]. We envision as one of the future targets of scaffolds loaded with osteogenic factors, antibiotics and/or mesenchymal stem cells delivery in the fracture area during the primary surgical treatment in order to decrease the complications rate. Summary of the discussed applications are summarized in Table 1.

## 5. Mathematical Modeling

Currently, mathematical modeling has gained a key role in the engineering and design of drug delivery systems. Models allow to rationalize and understand the most important involved phenomena that determine system behavior and, once their predictive capability has been assessed, they can be used to optimize the design of the device according to the desired performances. Starting from the seminal contribution of prof. Takeru Higuchi in 1961 [131] (Higuchi equation is currently used for some systems [132]) drug delivery modeling is still a growing field thanks, on one side, to the continuous development of new methods and to software optimization and increasing computational power on the other side. This allows to set-up complex simulations that can also involve, e.g., moving boundary problems or the integration of different physical models. In addition, drug delivery modeling takes advantage not only of standard approaches based on fundamental mass, energy and momentum conservation equations, but also of those methods focused on molecular scale that act as a “computational microscope”, which provide valuable insights not always accessible from an experimental point of view.

### 5.1. Modeling Approaches

As mentioned, drug delivery modeling embraces different approaches, each one with different accessible time and length scales, and thus with different purposes. Microscale models are aimed at understanding the fundamental interactions between drugs and their carrier or the target receptors, while macroscale models are commonly employed in order to optimize the device according to the specific needs. These two approaches can be also integrated in a multiscale modeling framework, where the effects of the specific interactions at molecular level are included in the macroscopic description of the system. The following paragraphs are intended to provide to the interested reader a general but comprehensive overview of the available modeling approaches employed and discussed in the literature. For each class of scaffolds presented in the previous sections, the most suitable computational method is discussed as well as the typical outcomes from calculations. Some relevant examples of the application of modeling to bone tissue engineering are also provided. 

#### 5.1.1. Microscale Modeling

Molecular dynamics (MD) simulations are currently a well-established and widely employed approach to investigate the system behavior at molecular scale. Atoms are represented as mutually interacting spheres, whose motion is computed by integrating Newton law [133]:

(1) $$\;m_{i}\frac{dr_{i}}{dt}\;=\;F_{i}\left(r\right)\;=\;-\nabla U\left(r\right)\;$$

where *m_i_* and *r_i_* are the mass and the coordinates of the *i*-th atom, respectively, *F_i_*(*r*) is the force acting on the *i*-th atom and *U*(*r*) is the potential energy; *F*(*r*) and *U*(*r*) depend only on atomic coordinates *r*. This constitutes a reasonable approximation when quantum effects are not relevant, i.e., when the motion of light species such as H_2_, D_2_ and He is not considered. 

**Figure 2:**
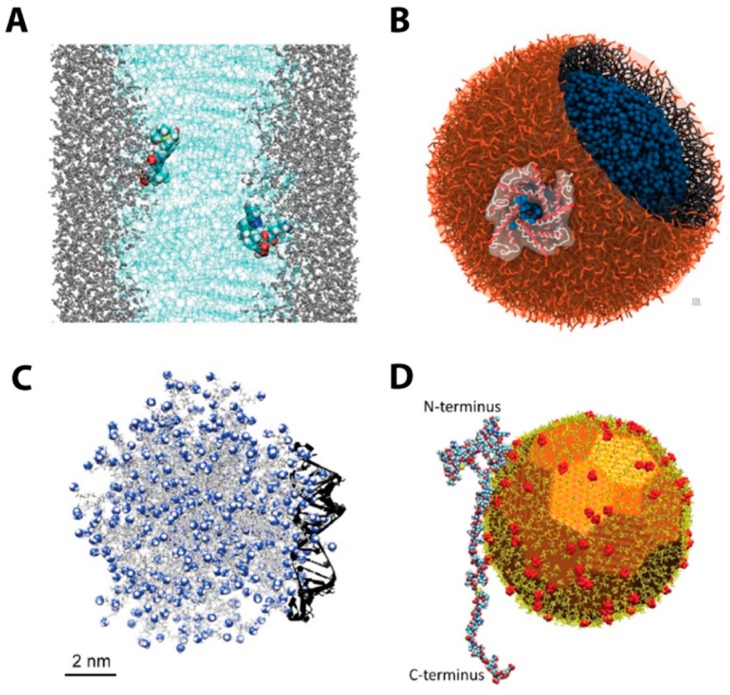
(**A**) Permeation of silatecan camptothecin drug analogue and a protonated 20(*S*)-4-aminobutyrate substituted prodrug in a hydrated dimyristoylphosphatidylcholine (DMPC) bilayer. Water molecules are represented as silver CPK, bilayer is represented as light blue lines and drugs are shown as VdW spheres. Reproduced with permission from Elsevier [155]. (**B**) Lipid vesicle containing a mechano-sensitive protein channel simulated with coarse-grain MARTINI force field. Water is represented as blue beads. Reproduced with permission from Royal Society of Chemistry [158]. (**C**) Binding between G7 PAMAM dendrimer and siRNA fragment; siRNA is represented as dark solid ribbons, while charged primary amine groups are represented as blue/white spheres. Reproduced with permission from Elsevier [150]. (**D**) Interaction between α-synuclein with 12 nm gold nanoparticle functionalized with partially ionized citrate ligands (charged moieties are shown as red spheres). Reproduced with permission from Wiley [161].

The potential energy function *U*(*r*) is usually called force field and accounts for several contributions, such as bonds, angles, dihedrals, electrostatic and Van der Waals interactions. Force fields are tested and parameterized according to high-level quantum chemistry calculations and experimental data. Literature offers many examples of general purposes force fields [134,135,136] as well as force fields tailored for a specific class of molecules, such as proteins [137], nucleic acids [138], lipids [139] and carbohydrates [140]. The typical length and time scales of MD simulations are nanometers and nanoseconds, although the microsecond time scale is becoming accessible thanks to the synergic effects of software optimization and the increasing computational capability [141] such as the use of Graphics Processing Units (GPUs). However, many phenomena of interest, such as protein folding or drug unbinding, take place on a time scale that is still out of reach for MD simulations (that is, from milliseconds to seconds). This issue can be overcome by employing enhanced sampling methods [142]. Briefly, an external bias potential is applied to the system in order to enhance the transition between metastable states separated by energy barriers whose magnitude is much higher than *k_B_T* (where *k_B_* is Boltzmann constant and *T* is the absolute temperature) and are thus rarely crossed in a standard simulation at constant temperature *T*. This also allows to recover the free energy of the system as a function of few relevant degrees of freedom, usually called collective variables (more details in Appendix). MD simulations are also ideal to investigate the non-covalent binding between nucleic acids and polycationic carriers, such as polyethyleneimine [143,144,145,146,147], polyarginine [145], polylysine [144,145,147] as well as dendrons of different generations [148,149,150]. Simulations at molecular level allow understanding the impact of parameters such as charge density and molecular flexibility. In addition, system behavior can be rationalized by computing a binding free energy with approaches such as MMPBSA [148] or enhanced sampling methods [145]. Recently, MD simulations started to be extensively employed for the simulation of lipid bilayers (which mimic cellular membranes but also liposomal carriers) and the permeation of drug molecules across the membrane (Figure 2C) [150]. Molecular simulations coupled with umbrella sampling (an enhanced sampling method) allow to obtain the free energy as a function of the distance between the centers of mass of the penetrant and the lipid bilayer [151,152,153,154] as a collective variable. This constitutes the input for the inhomogeneous solubility-diffusion model [155,156], with which diffusion coefficients and permeability as a function of the position into the membrane can be calculated, as well as average values (Figure 2A). Dickson and coworkers [152] employed MD-derived permeability values for seven active compounds in order to build a mechanistic macroscale model for membrane permeation. Lipid bilayers are also investigated by means of coarse-grain (CG) methods, where groups of atoms are embedded into beads (i.e., grouped in a single interaction center), which account for the main properties of the atoms that they include (hydrophilicity/hydrophobicity, charge, polarity). One of the most popular coarse-grain force fields, MARTINI [157], has four different main classes of beads: polar, non-polar, apolar and charged. As a matter of fact, MARTINI force field has been employed to simulate not only lipid membranes but also entire lipid particles (Figure 2B) [158,159]. Thanks to the detail at atomic scale provided by microscale models, systems like gold nanoparticles (Figure 2D) [160,161,162,163] have been also investigated through MD-based methods [164]. The aim of the simulations is to investigate the dynamic behavior at particle/environment interface, such as protein adsorption or the effect of decorating groups at particle surface. Other inorganic scaffolds and their interactions with proteins have been studied at a molecular level; a relevant example is constituted by the adsorption of Bone Morphogenetic Factor 2 (BMP2) on hydroxyapatite surfaces [165,166].

#### 5.1.2. Macroscale Modeling

Macroscale modeling is based on fundamental conservation equations and constitute a typical engineering tool for design and optimization. In the drug delivery field, they allow to account for the release of an active compound loaded in a matrix as well as the degradation of devices made of bioresorbable materials (like aliphatic polyesters), and their synergic effects. 

Indeed, degradation (if present) creates new diffusive paths that imply a dynamic increase of the diffusion coefficient of the drug and degradation products, which must be accounted for in a comprehensive modeling framework. In general, first-principle-based or mechanistic models require an understanding of the most important chemical and physical phenomena involved; they contain input parameters with a precise physical meaning (kinetic constants, diffusion coefficients, et cetera) that can be independently estimated from experimental data or suitable and validated correlations. Because of this, mechanistic models can be used for an optimal device design since they allow to make prediction for different operative conditions (that is, different from the ones used for model validation). Focusing on polymer-based devices, the main challenge is a proper description of transport phenomena within the device, since drug release can be due to different mechanisms and influenced by several factors [167,168] (water penetration, phase transition of polymeric excipients, molecular interactions with the device, changes in microenvironmental pH and ionic strength, et cetera); it is not possible to formulate a model that takes into account all involved phenomena and simplifications are an inescapable necessity to achieve a model that can be used for practical purposes. In most cases, diffusion can be assumed as Fickian, i.e., it can be described by means of Equation (A1); however, for other systems a non-Fickian diffusion is observed [169]. This is typical of glassy polymers (when the temperature is below the glass transition temperature *T_g_*), where the chains do not have enough mobility to allow an immediate diffusion of the solvent. As mentioned, diffusion coefficients must be properly estimated in order to achieve a reliable model. There are different theoretical approaches to account for complex environment like polymer matrices, which can be summarized as follows [169,170]:

- Obstruction theories: polymer chains are considered motionless if compared to solute and solvent molecules. Polymer chains are modeled as fixed impenetrable rods in solution that increase the mean diffusive path of the molecules;
- Hydrodynamic theories: this approach takes into account hydrodynamics interactions, like the frictional ones between the solute and the polymer, the solvent and the polymer and the solute and the solvent;
- Free volume theories: free volume is defined as the volume not occupied by matter or, more generally, as the volume of the system at a given temperature minus the volume of the same system at 0 K. Free volume rearrangements create pores and cavities where diffusing species can diffuse through. In other words, free volume is considered the main factor that determines molecular diffusion.

Additional efforts have been presented for hydrogels; apart from the diffusion coefficients dependent on water concentration mentioned for swelling controlled systems, different approaches have been discussed for polyelectrolyte hydrogels [171]. Amsden et al. [172] developed a model based on obstruction theories, which takes into account polymer ionization degree. Fatin-Rouge and coworkers [173] studied the motion of small ions in agarose hydrogels, accounting for steric and electrostatic interactions. Vega et al. [174] investigated ions diffusion in agarose hydrogels, proposing a model that explicitly underlines the effect of gel porosity and ions size. In this framework, microscale models can provide valuable insights concerning the effects of solute/matrix interactions and their impact on the observed release profile. In a recent work from Yan et al. [175], it has been observed that the initial pH of a hyaluronic acid hydrogel influenced the release rate of the Bone Morphogenetic Protein 2 loaded into the matrix. Molecular dynamics simulations allowed to highlight the presence of pH-dependent protein/hyaluronic acid interactions, which resulted in a relevant protein adsorption on hydrogel chain at pH 4.5 and a negligible one at pH 7. The understanding gained at molecular level has been included in a macroscale model, which could describe the observed release rate at different pH values. 

Focusing on some specific systems of interest, aliphatic polyesters are experiencing an ever-growing interest in the biomedical field, thanks to their degradation in vivo through hydrolysis (a second surgical operation to remove the device is not needed, improving patient care), biocompatibility (degradation products are metabolized by the human body itself) and versatility (material properties can be easily tailored for the specific application); therefore, it is not surprising to discover that literature offers a wide number of mathematical models for such systems [167,176,177,178,179,180,181]. This has been possible also because the main phenomena behind aliphatic polyesters degradation are now well-established and accepted in scientific literature [182]. Water diffuses into the polymer matrix (which exhibits a limited swelling) and starts breaking the ester bonds that constitute polymer backbone (focus in Appendix). Although the detail of the distribution is lost, the average properties of interest (molecular weights, polydispersity) can be easily obtained as a function of statistical moments. Also, in this framework there are models of different complexity [183,184,185,186,187]; Nishida et al. [184] obtained a simple equation for computing average molecular weights and polydispersity, accounting for autocatalysis but not for erosion. Casalini et al. [187] proposed a model that takes into account transport phenomena, autocatalysis, erosion and diffusion coefficients which depend on polymer number average molecular weight. Stochastic models describe hydrolysis as a random event; such an approach has been adopted, e.g., by Siepmann et al. [188] to describe drug release from microspheres made of poly(lactic-*co*-glycolic) acid. Diffusion coefficient depends on porosity, which increases in time and space because of hydrolysis. 

## 6. Conclusions

Skeletal conditions, such as fractures, osteomyelitis, osteoarthritis, osteonecrosis and bone cancer, affect a vast portion of the population, often requiring surgical procedures associated with extensive bone loss. Hence, in some cases, bone grafts are needed in order to restore the normal bone anatomy. Autografts and allografts are the current standards of treatment in such cases, but these approaches still display some non-negligible contraindications, like limited bone availablity for transplant or immunogenic reactions. Recent progress in material science allows reliable scaffolds to be exploited for bone repair. Several materials have already been studied both in vitro and in vivo, demonstrating promising results in terms of biocompatibility and biomechanical properties. Moreover, scaffolds for bone repair showed encouraging results when combined with drugs, growth factors and mesenchymal stem cells, which can co-operate facilitating de novo bone tissue deposition and mineralization. For this aim, the development of nanotechnology has a great potential, allowing to specifically target such therapeutic agents to the site of injured bone, avoiding systemic adverse reactions and reaching effective therapeutic effects with a low and steady dose. Although scaffolds for bone-targeted delivery demonstrated to be a valid approach to treat a large amount of bone diseases, some critical issues should still be investigated. In fact, due to the limited literature in humans, some concerns are still open, like a better understanding of nanotoxicity, drug loading capacity and delivering, and possible long-term adverse effects of such implants. Considering the vast amount of literature showing the positive implications of such an approach in treating bone conditions, more extensive studies of novel biomaterials and their clinical applications are encouraged.

## Figures and Tables

**Table 1:** Clinical applications in drug delivery.

Disease	Therapeutic Agent	Drug Delivery System	Main Outcome
Osteomyelitis	Antibiotics	PMMA, PLGA	Releases high levels of antibiotic at a local administration site. No side effects.
Cancer bone metastasis	DXR	PLGA-ALE	Higher or equal efficacy than free DXR in prevention of osteolytic bone metastases and reduction of DXR concentration in healthy tissues.
	PTX, ALN	PEG	Marked increase in their half-life. Great binding affinity to the bone in vitro.
Osteosarcoma	DXR	PLGA	Enhance DXR antitumoral efficacy compared with free drug.
Osteoarthritis	Dextran	Cationic nanoparticles	Increases the retention time, maintaining cartilage structure and composition.
	IL-1Ra	IL-1Ra-tethered nanoparticles	
Osteonecrosis	Simvastatin	PDLLA, PLGA	Decrease of inflammation. Facilitates osteogenic differentiation and maturation.
	PDGF		Decrease of inflammation. Cell recruitment, (imitating the early mitogenic stage in wound healing).
Delayed-non unions	Osteoinductive agents, antibiotics	Composite systems	Promotes fracture healing and decreases risk of secondary osteomyelitis.

## Appendix A. On Mathematical Modeling

### Appendix A.1. Microscale Modeling

MD simulations do not allow to study chemical reactions (electrons are not explicitly taken into account) but they provide valuable insights for those systems where non-covalent interactions are fundamental. In addition, solvent molecules and ions are explicitly included and their role is accounted for in detail. For the sake of completeness, it is possible to study chemical reactions through hybrid quantum mechanics/molecular mechanics (QM/MM) methods, e.g., when dealing with the covalent binding of an inhibitor to an enzyme; an extensive discussion on this complex topic is beyond the purpose of this paper, and the interested readers are referred to dedicated reviews [189,190]. A typical application of MD simulation is constituted by the study of the non-covalent binding between a drug molecule (the ligand) and the target protein (the receptor) [190]. When the crystallographic structure of the complex is available, simulations allow understanding the most relevant interactions that lead to binding and the specific amino acids involved. In addition, the affinity between the receptor and the ligand can be quantitatively estimated by computing the binding free energy *ΔG_bind_*. If potential binding sites of the target protein are known, a preliminary in silico screening can be performed in order to evaluate the affinity of potential ligands. The initial step is usually constituted by *docking*, where binding poses are evaluated by computing the binding free energy as a function of coordinates by means of an empirical formula [191]. The investigated compounds are ranked according to the obtained affinity values. Docking is an efficient but simplified approach (receptor and ligand flexibility are not always considered as well as entropic contributions, solvent and ions are implicitly accounted for) that allows to discriminate between binders and non-binders, but not between weak and strong binders.

The obtained binding poses can be used as input for molecular dynamics simulations, where binding is described as a dynamic process and solvent and ions are accounted for; *ΔG_bind_* values can be calculated with different methods such as Linear Interaction Energy (LIE) [192], Molecular Mechanics Generalized Born Surface Area (MMGBSA) [193], Molecular Mechanics Poisson Boltzmann Surface Area (MMPBSA) [193] and Alchemic Perturbation (AP) [194]. Currently, MMPBSA and MMGBSA represent a good compromise between efficiency and accuracy, at least for ranking similar ligands after docking [195]. AP and enhanced sampling methods provide a more accurate estimation of the affinity, but they are seldom used in the initial screening since they are demanding from a computational point of view.

### Appendix A.2. Macroscale Modeling: Diffusion

#### Concerning Diffusion Mechanisms

- Diffusion controlled systems: drug release is determined by the concentration gradient between the loaded device and the external environment. The starting point for the description of such systems is the Fick’s second law:
  (A1) $$\;\frac{\partial C}{\partial t}=\nabla \left(D\nabla C\right)\;$$
  where *C* is drug concentration in the device, *t* is time and *D* is the diffusion coefficient; this parameter depends on several factors (drug, environment, temperature, et cetera) and accounts for the ability of the loaded compound to move within the device. For simple geometries (slabs, cylinders, spheres) and simplified cases (diffusion coefficient constant in time and space, initial uniform drug distribution, and so on) analytical solutions of Equation (A1) are available in literature [196]. The challenge is a robust estimation of the diffusion coefficient in a complex environment such as polymer matrices, as explained below;
- Swelling controlled systems: in systems such as hydrogels, the swelling of the polymer matrix can be the rate determining step for drug release. When the starting point is constituted by a dry (non-swollen) polymer matrix, the drug is embedded in a dense network where the mobility of polymer chains and active compound is strongly hindered. As soon as water starts to penetrate into the matrix, polymer chains relax (their mobility increases) and device volume increases. This dramatically changes the conditions for drug transport from the dry and swollen matrix, since the active molecules become free to move towards the external environment. In this framework, modeling approaches take into account at the same time water penetration into the matrix and drug diffusion, with time and spatial dependent diffusion coefficients that are function of local water concentration. In addition, since matrix volume increases in time because of water penetration, the model has moving boundary conditions that must be properly accounted for [197,198];
- Degradation and erosion controlled systems: as already mentioned, the hydrolysis of the matrix can promote drug release because of the dynamic increase of the diffusion coefficient. Water penetrates into the matrix and starts breaking the backbone of long water-insoluble chains in smaller fragments. In this framework, the meaning of “degradation” and “erosion” must be properly specified. Degradation is the chain scission process, while erosion is the mass loss from the bulk due to the diffusion of the small water-soluble oligomers. The dynamics of water penetration and hydrolysis are determinant. If water penetration is much faster than hydrolysis, water concentration is approximatively uniform into the device volume, which is in turn experiences a uniform degradation; this situation is usually referred as bulk or homogeneous degradation.

On the other hand, when water consumption is faster than its diffusion within the polymer, the device experiences degradation and erosion only at the surface while its core remains intact; this is referred as surface or heterogeneous degradation. A suitable modeling approach must contain both degradation kinetics and transport phenomena (in order to take into account the different degradation mechanisms) as well as diffusion coefficients that depend on polymer degradation. In addition, while the volume of bulk degrading matrices usually remains constant at least during the time interval needed for the complete release, surface eroding matrices experience volume decrease, which imply a moving boundary problem [199,200].

### Appendix A.3. Macroscale Modeling: Degradation

The interplay between the time scales for water diffusion and consumption determine whether bulk or surface hydrolysis occurs. The resulting oligomers cause a decrease in the microenvironmental pH, because of the dissociation of their carboxylic terminal; this, in turn, enhance hydrolysis rate which is catalyzed in acid environments, thus leading to an autocatalytic degradation mechanism. If oligomer concentration is high in the bulk of the device because of diffusive limitations, bulk degradation can become inhomogeneous because the device core degrades faster, due to the lower local pH. In addition, degradation creates new and wider diffusive paths, which implies a dynamic increase of the diffusion coefficient of each specie, as previously mentioned. A comprehensive modeling approach for aliphatic polyesters must combine degradation kinetics with transport phenomena, where diffusion coefficients are not constant but they depend on the extent of degradation. In this framework, the purpose of the model is twofold: on one side, it allows determining drug release rate as a function of time, taking into account the synergic effects of degradation on diffusion. On the other side, it is possible to characterize the decrease of number and weight average molecular weight over time, which is extremely important in order to assure, e.g., the desired mechanical properties over a determined time span. The existing mechanistic models can be divided in two categories: deterministic and stochastic. Deterministic models employ deterministic equations to characterize polymer degradation. Although models of different complexity have been proposed in literature, the starting point is usually constituted by mass balances. A simple model has been proposed by Antheunis and coworkers [201] where polymer degradation is described considering the concentration of ester bonds; transport phenomena (i.e., water and oligomers diffusion) and mass loss are not included but autocatalysis is accounted for. Rothstein et al. [202] developed a model for both bulk and surface eroding polymer, taking into account transport phenomena and a porosity-dependent diffusion coefficient. However, autocatalysis is not included. A common approach for a detailed modeling is constitute by population balances, where a mass balance for each chain composed by n repeating units is written. Since this approach is computationally expensive (writing a mass balance for each chain length value can lead to a system of 10^5^ differential equations), the resulting model is usually solved through the method of statistical moments [203].

## References

[1] Carrington J.L. (2005). Aging bone and cartilage: Cross-cutting issues. Biochem. Biophys. Res. Commun..

[2] Olshansky S.J., Passaro D.J., Hershow R.C., Layden J., Carnes B.A., Brody J., Hayflick L., Butler R.N., Allison D.B., Ludwig D.S. (2005). A potential decline in life expectancy in the united states in the 21st century. N. Engl. J. Med..

[3] Cancedda R., Dozin B., Giannoni P., Quarto R. (2003). Tissue engineering and cell therapy of cartilage and bone. Matrix Biol..

[4] Gao C., Deng Y., Feng P., Mao Z., Li P., Yang B., Deng J., Cao Y., Shuai C., Peng S. (2014). Current progress in bioactive ceramic scaffolds for bone repair and regeneration. Int. J. Mol. Sci..

[5] Bauer T.W., Muschler G.F. (2000). Bone graft materials. An overview of the basic science. Clin. Orthop. Relat. Res..

[6] Dimitriou R., Mataliotakis G.I., Angoules A.G., Kanakaris N.K., Giannoudis P.V. (2011). Complications following autologous bone graft harvesting from the iliac crest and using the ria: A systematic review. Injury.

[7] Shafiei Z., Bigham A.S., Dehghani S.N., Nezhad S.T. (2009). Fresh cortical autograft versus fresh cortical allograft effects on experimental bone healing in rabbits: Radiological, histopathological and biomechanical evaluation. Cell Tissue Bank..

[8] Suchomel P., Barsa P., Buchvald P., Svobodnik A., Vanickova E. (2004). Autologous versus allogenic bone grafts in instrumented anterior discectomy and fusion: A perspective with respect to bone union pattern. Eur. J. Spine.

[9] Hench L.L., Polak J.M. (2002). Third-generation biomedical materials. Science.

[10] Williams D.F. (2008). On the mechanisms of biocompatibility. Biomaterials.

[11] Rouwkema J., Rivron N.C., van Blitterswijk C.A. (2008). Vascularization in tissue engineering. Trends Biotechnol..

[12] Tu J., Wang H., Li H., Dai K., Wang J., Zhang X. (2009). The in vivo bone formation by mesenchymal stem cells in zein scaffolds. Biomaterials.

[13] Murphy C.M., Haugh M.G., O’Brien F.J. (2010). The effect of mean pore size on cell attachment, proliferation and migration in collagen-glycosaminoglycan scaffolds for bone tissue engineering. Biomaterials.

[14] Gu W., Wu C., Chen J., Xiao Y. (2013). Nanotechnology in the targeted drug delivery for bone diseases and bone regeneration. Int. J. Nanomed..

[15] Fuchs J.R., Nasseri B.A., Vacanti J.P. (2001). Tissue engineering: A 21st century solution to surgical reconstruction. Ann. Thorac. Surg..

[16] Kretlow J.D., Mikos A.G. (2007). Review: Mineralization of synthetic polymer scaffolds for bone tissue engineering. Tissue Eng..

[17] Ishaug S.L., Yaszemski M.J., Bizios R., Mikos A.G. (1994). Osteoblast function on synthetic biodegradable polymers. J. Biomed. Mater. Res..

[18] Ali Akbari Ghavimi S., Ebrahimzadeh M.H., Solati-Hashjin M., Abu Osman N.A. (2015). Polycaprolactone/starch composite: Fabrication, structure, properties, and applications. J. Biomed. Mater. Res. A.

[19] Athanasiou K.A., Agrawal C.M., Barber F.A., Burkhart S.S. (1998). Orthopaedic applications for pla-pga biodegradable polymers. Arthroscopy.

[20] Ghassemi T., Shahroodi A., Ebrahimzadeh M.H., Mousavian A., Movaffagh J., Moradi A. (2018). Current concepts in scaffolding for bone tissue engineering. Arch. Bone Jt. Surg..

[21] Yan J., Li J., Runge M.B., Dadsetan M., Chen Q., Lu L., Yaszemski M.J. (2011). Cross-linking characteristics and mechanical properties of an injectable biomaterial composed of polypropylene fumarate and polycaprolactone co-polymer. J. Biomater. Sci. Polym. Ed..

[22] Moradi A., Ataollahi F., Sayar K., Pramanik S., Chong P.P., Khalil A.A., Kamarul T., Pingguan-Murphy B. (2016). Chondrogenic potential of physically treated bovine cartilage matrix derived porous scaffolds on human dermal fibroblast cells. J. Biomed. Mater. Res. A.

[23] Pei M., Li J.T., Shoukry M., Zhang Y. (2011). A review of decellularized stem cell matrix: A novel cell expansion system for cartilage tissue engineering. Eur. Cell Mater..

[24] Yarlagadda P.K., Chandrasekharan M., Shyan J.Y. (2005). Recent advances and current developments in tissue scaffolding. Biomed. Mater. Eng..

[25] Russo E., Gaglianone N., Baldassari S., Parodi B., Cafaggi S., Zibana C., Donalisio M., Cagno V., Lembo D., Caviglioli G. (2014). Preparation, characterization and in vitro antiviral activity evaluation of foscarnet-chitosan nanoparticles. Colloids Surf. B Biointerfaces.

[26] Cao L., Werkmeister J.A., Wang J., Glattauer V., McLean K.M., Liu C. (2014). Bone regeneration using photocrosslinked hydrogel incorporating rhbmp-2 loaded 2-n, 6-o-sulfated chitosan nanoparticles. Biomaterials.

[27] Cheng H., Chawla A., Yang Y., Li Y., Zhang J., Jang H.L., Khademhosseini A. (2017). Development of nanomaterials for bone-targeted drug delivery. Drug Discov. Today.

[28] Battaglia L., Gallarate M. (2012). Lipid nanoparticles: State of the art, new preparation methods and challenges in drug delivery. Expert Opin. Drug Deliv..

[29] Dumas A., Gaudin-Audrain C., Mabilleau G., Massin P., Hubert L., Baslè M.F., Chappard D. (2006). The influence of processes for the purification of human bone allografts on the matrix surface and cytocompatibility. Biomaterials.

[30] Winckler H., Haiden P. (2017). Allograft bone as antibiotic carrier. J. Bone Jt. Infect..

[31] Wang Y., Zhao Q., Han N., Bai L., Li J., Liu J., Che E., Hu L., Zhang Q., Jiang T. (2015). Mesoporous silica nanoparticles in drug delivery and biomedical applications. Nanomedicine.

[32] Cabuzu D., Cirja A., Puiu R., Grumezescu A.M. (2015). Biomedical applications of gold nanoparticles. Curr. Top. Med. Chem..

[33] Sul O.J., Kim J.C., Kyung T.W., Kim H.J., Kim Y.Y., Kim S.H., Kim J.S., Choi H.S. (2010). Gold nanoparticles inhibited the receptor activator of nuclear factor-kappab ligand (rankl)-induced osteoclast formation by acting as an antioxidant. Biosci. Biotechnol. Biochem..

[34] Ghosh P., Han G., De M., Kim C.K., Rotello V.M. (2008). Gold nanoparticles in delivery applications. Adv. Drug Deliv. Rev..

[35] Yi C., Liu D., Fong C.C., Zhang J., Yang M. (2010). Gold nanoparticles promote osteogenic differentiation of mesenchymal stem cells through p38 mapk pathway. ACS Nano.

[36] Zhang Q., Mochalin V.N., Neitzel I., Knoke I.Y., Han J., Klug C.A., Zhou J.G., Lelkes P.I., Gogotsi Y. (2011). Fluorescent plla-nanodiamond composites for bone tissue engineering. Biomaterials.

[37] Biltz R.M., Pellegrino E.D. (1969). The chemical anatomy of bone. I. A comparative study of bone composition in sixteen vertebrates. J. Bone Jt. Surg. Am..

[38] Bose S., Tarafder S. (2012). Calcium phosphate ceramic systems in growth factor and drug delivery for bone tissue engineering: A review. Acta Biomater..

[39] Dorozhkin S.V., Epple M. (2002). Biological and medical significance of calcium phosphates. Angew. Chem. Int. Ed. Engl..

[40] Ambre A.H., Katti D.R., Katti K.S. (2015). Biomineralized hydroxyapatite nanoclay composite scaffolds with polycaprolactone for stem cell-based bone tissue engineering. J. Biomed. Mater. Res. A.

[41] Fielding G.A., Bandyopadhyay A., Bose S. (2012). Effects of silica and zinc oxide doping on mechanical and biological properties of 3d printed tricalcium phosphate tissue engineering scaffolds. Dent. Mater..

[42] Alves Cardoso D., Jansen J.A., Leeuwenburgh S.C. (2012). Synthesis and application of nanostructured calcium phosphate ceramics for bone regeneration. J. Biomed. Mater. Res. B.

[43] Athanasiou V.T., Papachristou D.J., Panagopoulos A., Saridis A., Scopa C.D., Megas P. (2010). Histological comparison of autograft, allograft-dbm, xenograft, and synthetic grafts in a trabecular bone defect: An experimental study in rabbits. Med. Sci. Monit..

[44] Datta A., Gheduzzi S., Miles A.W. (2006). A comparison of the viscoelastic properties of bone grafts. Clin. Biomech..

[45] Capanna V., Milano G., Pagano E., Barba M., Cicione C., Salonna G., Lattanzi W., Logroscino G. (2014). Bone substitutes in orthopaedic surgery: From basic science to clinical practice. J. Mater. Sci. Mater. Med..

[46] Knofler W., Barth T., Graul R., Krampe D. (2016). Retrospective analysis of 10,000 implants from insertion up to 20 years-analysis of implantations using augmentative procedures. Int. J. Implant Dent..

[47] Ceccarelli G., Presta R., Benedetti L., Gabriella M., De Angelis C., Marco Lupi S., Rodriguez y Baena R. (2017). Emerging perspectives in scaffold for tissue engineering in oral surgery. Stem. Cells Int..

[48] Colaço H.B., Shah Z., Back D., Davies A., Ajuied A. (2015). Xenograft in orthopaedics. Orthop. Trauma.

[49] Pertici G., Rossi F., Casalini T., Perale G. (2014). Composite polymer-coated mineral grafts for bone regeneration: Material characterisation and model study. Ann. Oral Maxillofac. Surg..

[50] Stacchi C., Lombardi T., Perinetti G., Traini T. (2018). New bone formation after transcrestal sinus floor elevation was influenced by sinus cavity dimensions: A prospective histologic and histomorphometric study. Clin. Oral. Implants Res..

[51] Rossi F., Santoro M., Perale G. (2015). Polymeric scaffolds as stem cell carriers in bone repair. J. Tissue Eng. Regen. Med..

[52] D’Alessandro D., Perale G., Milazzo M., Moscato S., Stefanini C., Pertici G., Danti S. (2017). Bovine bone matrix/poly(l-lactic-co-e-caprolactone)/gelatin hybrid scaffold (smartbone1) for maxillary sinus augmentation: A histologic study on bone regeneration. Int. J. Pharm..

[53] Roato I., Belisario D.C., Compagno M., Verderio L., Sighinolfi A., Mussano F., Genova T., Veneziano F., Pertici G., Perale G. (2018). Adipose-derived stromal vascular fraction/xenohybrid bone scaffold: An alternative source for bone regeneration. Stem. Cells Int..

[54] Mbalaviele G., Sheikh S., Stains J.P., Salazar V.S., Cheng S.L., Chen D., Civitelli R. (2005). Beta-catenin and bmp-2 synergize to promote osteoblast differentiation and new bone formation. J. Cell Biochem..

[55] Vahle J.L., Sato M., Long G.G., Young J.K., Francis P.C., Engelhardt J.A., Westmore M.S., Linda Y., Nold J.B. (2002). Skeletal changes in rats given daily subcutaneous injections of recombinant human parathyroid hormone (1-34) for 2 years and relevance to human safety. Toxicol. Pathol..

[56] Pilitsis J.G., Lucas D.R., Rengachary S.S. (2002). Bone healing and spinal fusion. Neurosurg. Focus.

[57] Hosain F., Spencer R.P., Couthon H.M., Sturtz G.L. (1996). Targeted delivery of antineoplastic agent to bone: Biodistribution studies of technetium-99m-labeled gem-bisphosphonate conjugate of methotrexate. J. Nucl. Med..

[58] Lo K.W., Ashe K.M., Kan H.M., Laurencin C.T. (2012). The role of small molecules in musculoskeletal regeneration. Regen. Med..

[59] Hoffman M.D., Benoit D.S. (2015). Agonism of wnt-beta-catenin signalling promotes mesenchymal stem cell (msc) expansion. J. Tissue Eng. Regen. Med..

[60] Benoit D.S., Nuttelman C.R., Collins S.D., Anseth K.S. (2006). Synthesis and characterization of a fluvastatin-releasing hydrogel delivery system to modulate hmsc differentiation and function for bone regeneration. Biomaterials.

[61] Murata K., Ito H., Yoshitomi H., Yamamoto K., Fukuda A., Yoshikawa J., Furu M., Ishikawa M., Shibuya H., Matsuda S. (2014). Inhibition of mir-92a enhances fracture healing via promoting angiogenesis in a model of stabilized fracture in young mice. J. Bone Miner. Res..

[62] Wang Y., Malcolm D.W., Benoit D.S.W. (2017). Controlled and sustained delivery of sirna/nps from hydrogels expedites bone fracture healing. Biomaterials.

[63] Lieberman J.R., Daluiski A., Einhorn T.A. (2002). The role of growth factors in the repair of bone. Biology and clinical applications. J. Bone Jt. Surg. Am..

[64] Ripamonti U., Reddi A.H. (1992). Growth and morphogenetic factors in bone induction: Role of osteogenin and related bone morphogenetic proteins in craniofacial and periodontal bone repair. Crit. Rev. Oral. Biol. Med..

[65] Ratko T.A., Belinson S.E., Samson D.J., Bonnell C., Ziegler K.M., Aronson N. (2010). Bone Morphogenetic Protein: The State of the Evidence of on-Label and Off-Label Use.

[66] Haidar Z.S., Hamdy R.C., Tabrizian M. (2009). Delivery of recombinant bone morphogenetic proteins for bone regeneration and repair. Part A: Current challenges in bmp delivery. Biotechnol. Lett..

[67] Jones A.L., Bucholz R.W., Bosse M.J., Mirza S.K., Lyon T.R., Webb L.X., Pollak A.N., Golden J.D., Valentin-Opran A. (2006). Recombinant human bmp-2 and allograft compared with autogenous bone graft for reconstruction of diaphyseal tibial fractures with cortical defects. A randomized, controlled trial. J. Bone Jt. Surg. Am..

[68] Emara K.M., Diab R.A., Emara A.K. (2015). Recent biological trends in management of fracture non-union. World J. Orthop..

[69] Boraiah S., Paul O., Hawkes D., Wickham M., Lorich D.G. (2009). Complications of recombinant human bmp-2 for treating complex tibial plateau fractures: A preliminary report. Clin. Orthop. Relat. Res..

[70] Kim H.K., Shim W.S., Kim S.E., Lee K.H., Kang E., Kim J.H., Kim K., Kwon I.C., Lee D.S. (2009). Injectable in situ-forming ph/thermo-sensitive hydrogel for bone tissue engineering. Tissue Eng. Part A.

[71] Andreassen T.T., Ejersted C., Oxlund H. (1999). Intermittent parathyroid hormone (1–34) treatment increases callus formation and mechanical strength of healing rat fractures. J. Bone Miner. Res..

[72] Jung R.E., Cochran D.L., Domken O., Seibl R., Jones A.A., Buser D., Hammerle C.H. (2007). The effect of matrix bound parathyroid hormone on bone regeneration. Clin. Oral. Implants Res..

[73] Kothari R., Kumar V., Jena R., Tunga R., Tunga B.S. (2011). Modes of degradation and impurity characterization in rhpth (1–34) during stability studies. PDA J. Pharm. Sci. Technol..

[74] Zhang J., Tu Q., Bonewald L.F., He X., Stein G., Lian J., Chen J. (2011). Effects of mir-335-5p in modulating osteogenic differentiation by specifically downregulating wnt antagonist dkk1. J. Bone Miner. Res..

[75] Lietman S.A., Ding C., Cooke D.W., Levine M.A. (2005). Reduction in gsalpha induces osteogenic differentiation in human mesenchymal stem cells. Clin. Orthop. Relat. Res..

[76] Nelson C.E., Kim A.J., Adolph E.J., Gupta M.K., Yu F., Hocking K.M., Davidson J.M., Guelcher S.A., Duvall C.L. (2014). Tunable delivery of sirna from a biodegradable scaffold to promote angiogenesis in vivo. Adv. Mater..

[77] Semple S.C., Akinc A., Chen J., Sandhu A.P., Mui B.L., Cho C.K., Sah D.W., Stebbing D., Crosley E.J., Yaworski E. (2010). Rational design of cationic lipids for sirna delivery. Nat. Biotechnol..

[78] Xue H.Y., Liu S., Wong H.L. (2014). Nanotoxicity: A key obstacle to clinical translation of sirna-based nanomedicine. Nanomedicine.

[79] Whitehead K.A., Langer R., Anderson D.G. (2009). Knocking down barriers: Advances in sirna delivery. Nat. Rev. Drug Discov..

[80] Zintchenko A., Philipp A., Dehshahri A., Wagner E. (2008). Simple modifications of branched pei lead to highly efficient sirna carriers with low toxicity. Bioconjug. Chem..

[81] Nguyen K., Dang P.N., Alsberg E. (2013). Functionalized, biodegradable hydrogels for control over sustained and localized sirna delivery to incorporated and surrounding cells. Acta Biomater..

[82] Singha K., Namgung R., Kim W.J. (2011). Polymers in small-interfering rna delivery. Nucl. Acid Ther..

[83] Convertine A.J., Benoit D.S., Duvall C.L., Hoffman A.S., Stayton P.S. (2009). Development of a novel endosomolytic diblock copolymer for sirna delivery. J. Control Release.

[84] Malcolm D.W., Freeberg M.A.T., Wang Y., Sims K.R., Awad H.A., Benoit D.S.W. (2017). Diblock copolymer hydrophobicity facilitates efficient gene silencing and cytocompatible nanoparticle-mediated sirna delivery to musculoskeletal cell types. Biomacromolecules.

[85] Laurencin C.T., Ashe K.M., Henry N., Kan H.M., Lo K.W. (2014). Delivery of small molecules for bone regenerative engineering: Preclinical studies and potential clinical applications. Drug Discov. Today.

[86] Li Y.F., Luo E., Feng G., Zhu S.S., Li J.H., Hu J. (2010). Systemic treatment with strontium ranelate promotes tibial fracture healing in ovariectomized rats. Osteoporos. Int..

[87] Tai I.C., Fu Y.C., Wang C.K., Chang J.K., Ho M.L. (2013). Local delivery of controlled-release simvastatin/plga/hap microspheres enhances bone repair. Int. J. Nanomed..

[88] Mundy G., Garrett R., Harris S., Chan J., Chen D., Rossini G., Boyce B., Zhao M., Gutierrez G. (1999). Stimulation of bone formation in vitro and in rodents by statins. Science.

[89] Ozec I., Kilic E., Gumus C., Goze F. (2007). Effect of local simvastatin application on mandibular defects. J. Craniofac. Surg..

[90] Bradley J.D., Cleverly D.G., Burns A.M., Helm N.B., Schmid M.J., Marx D.B., Cullen D.M., Reinhardt R.A. (2007). Cyclooxygenase-2 inhibitor reduces simvastatin-induced bone morphogenetic protein-2 and bone formation in vivo. J. Periodontal. Res..

[91] Calixto J.C., Lima C.E., Frederico L., Lima R.P., Anbinder A.L. (2011). The influence of local administration of simvastatin in calvarial bone healing in rats. J. Craniomaxillofac. Surg..

[92] Stein D., Lee Y., Schmid M.J., Killpack B., Genrich M.A., Narayana N., Marx D.B., Cullen D.M., Reinhardt R.A. (2005). Local simvastatin effects on mandibular bone growth and inflammation. J. Periodontol..

[93] Lauing K.L., Sundaramurthy S., Nauer R.K., Callaci J.J. (2014). Exogenous activation of wnt/beta-catenin signaling attenuates binge alcohol-induced deficient bone fracture healing. Alcohol. Alcohol..

[94] Macsai C.E., Foster B.K., Xian C.J. (2008). Roles of wnt signalling in bone growth, remodelling, skeletal disorders and fracture repair. J. Cell Physiol..

[95] Bernick J., Wang Y., Sigal I.A., Alman B.A., Whyne C.M., Nam D. (2014). Parameters for lithium treatment are critical in its enhancement of fracture-healing in rodents. J. Bone Jt. Surg. Am..

[96] Kulkarni N.H., Onyia J.E., Zeng Q., Tian X., Liu M., Halladay D.L., Frolik C.A., Engler T., Wei T., Kriauciunas A. (2006). Orally bioavailable gsk-3alpha/beta dual inhibitor increases markers of cellular differentiation in vitro and bone mass in vivo. J. Bone Miner. Res..

[97] Sisask G., Marsell R., Sundgren-Andersson A., Larsson S., Nilsson O., Ljunggren O., Jonsson K.B. (2013). Rats treated with azd2858, a gsk3 inhibitor, heal fractures rapidly without endochondral bone formation. Bone.

[98] Wang Y., Newman M.R., Ackun-Farmmer M., Baranello M.P., Sheu T.J., Puzas J.E., Benoit D.S.W. (2017). Fracture-targeted delivery of beta-catenin agonists via peptide-functionalized nanoparticles augments fracture healing. ACS Nano.

[99] Baron R., Hesse E. (2012). Update on bone anabolics in osteoporosis treatment: Rationale, current status, and perspectives. J. Clin. Endocrinol. Metab..

[100] Kondiah P.J., Choonara Y.E., Kondiah P.P., Marimuthu T., Kumar P., du Toit L.C., Pillay V. (2016). A review of injectable polymeric hydrogel systems for application in bone tissue engineering. Molecules.

[101] Lucke M., Schmidmaier G., Sadoni S., Wildemann B., Schiller R., Stemberger A., Haas N.P., Raschke M. (2003). A new model of implant-related osteomyelitis in rats. J. Biomed. Mater. Res. B.

[102] Koort J.K., Makinen T.J., Suokas E., Veiranto M., Jalava J., Knuuti J., Tormala P., Aro H.T. (2005). Efficacy of ciprofloxacin-releasing bioabsorbable osteoconductive bone defect filler for treatment of experimental osteomyelitis due to staphylococcus aureus. Antimicrob. Agents Chemother..

[103] Dorati R., DeTrizio A., Modena T., Conti B., Benazzo F., Gastaldi G., Genta I. (2017). Biodegradable scaffolds for bone regeneration combined with drug-delivery systems in osteomyelitis therapy. Pharmaceuticals.

[104] Morgenstern M., Post V., Erichsen C., Hungerer S., Buhren V., Militz M., Richards R.G., Moriarty T.F. (2016). Biofilm formation increases treatment failure in staphylococcus epidermidis device-related osteomyelitis of the lower extremity in human patients. J. Orthop. Res..

[105] Ma T., Shang B.C., Tang H., Zhou T.H., Xu G.L., Li H.L., Chen Q.H., Xu Y.Q. (2011). Nano-hydroxyapatite/chitosan/konjac glucomannan scaffolds loaded with cationic liposomal vancomycin: Preparation, in vitro release and activity against staphylococcus aureus biofilms. J. Biomater. Sci. Polym. Ed..

[106] Peng K.T., Chen C.F., Chu I.M., Li Y.M., Hsu W.H., Hsu R.W., Chang P.J. (2010). Treatment of osteomyelitis with teicoplanin-encapsulated biodegradable thermosensitive hydrogel nanoparticles. Biomaterials.

[107] Salerno M., Cenni E., Fotia C., Avnet S., Granchi D., Castelli F., Micieli D., Pignatello R., Capulli M., Rucci N. (2010). Bone-targeted doxorubicin-loaded nanoparticles as a tool for the treatment of skeletal metastases. Curr. Cancer Drug Targets.

[108] Coleman R.E. (2006). Clinical features of metastatic bone disease and risk of skeletal morbidity. Clin. Cancer Res..

[109] Lipton A. (2008). Emerging role of bisphosphonates in the clinic—Antitumor activity and prevention of metastasis to bone. Cancer Treat. Rev..

[110] Li C.J., Liu X.Z., Zhang L., Chen L.B., Shi X., Wu S.J., Zhao J.N. (2016). Advances in bone-targeted drug delivery systems for neoadjuvant chemotherapy for osteosarcoma. Orthop. Surg..

[111] Clezardin P., Benzaid I., Croucher P.I. (2011). Bisphosphonates in preclinical bone oncology. Bone.

[112] Coleman R.E., McCloskey E.V. (2011). Bisphosphonates in oncology. Bone.

[113] Clementi C., Miller K., Mero A., Satchi-Fainaro R., Pasut G. (2011). Dendritic poly(ethylene glycol) bearing paclitaxel and alendronate for targeting bone neoplasms. Mol. Pharm..

[114] Elazar V., Adwan H., Bauerle T., Rohekar K., Golomb G., Berger M.R. (2010). Sustained delivery and efficacy of polymeric nanoparticles containing osteopontin and bone sialoprotein antisenses in rats with breast cancer bone metastasis. Int. J. Cancer.

[115] Reufsteck C., Lifshitz-Shovali R., Zepp M., Bauerle T., Kubler D., Golomb G., Berger M.R. (2012). Silencing of skeletal metastasis-associated genes impairs migration of breast cancer cells and reduces osteolytic bone lesions. Clin. Exp. Metastasis.

[116] Morrow J.J., Khanna C. (2015). Osteosarcoma genetics and epigenetics: Emerging biology and candidate therapies. Crit. Rev. Oncog..

[117] Hendershot E., Volpe J., Taylor T., Nicksy D., Mills D., Ramachandran N., Shaikh F., Riss V., Grant R., Gupta A. (2018). Outpatient high-dose methotrexate for osteosarcoma: It’s safe and feasible, if you want it. J. Pediatr. Hematol. Oncol..

[118] Zhou W., Hao M., Du X., Chen K., Wang G., Yang J. (2014). Advances in targeted therapy for osteosarcoma. Discov. Med..

[119] Danhier F., Ansorena E., Silva J.M., Coco R., Le Breton A., Preat V. (2012). Plga-based nanoparticles: An overview of biomedical applications. J. Control Release.

[120] Taruc-Uy R.L., Lynch S.A. (2013). Diagnosis and treatment of osteoarthritis. Prim. Care.

[121] Blanco F.J. (2014). Osteoarthritis: Something is moving. Reumatol. Clin..

[122] Morgen M., Tung D., Boras B., Miller W., Malfait A.M., Tortorella M. (2013). Nanoparticles for improved local retention after intra-articular injection into the knee joint. Pharm. Res..

[123] Whitmire R.E., Wilson D.S., Singh A., Levenston M.E., Murthy N., Garcia A.J. (2012). Self-assembling nanoparticles for intra-articular delivery of anti-inflammatory proteins. Biomaterials.

[124] Hong S.H., Shetty A.A., Kim S.J., Kim Y.S., Choi N.Y., Kim N.H. (2013). Treatment of osteonecrosis in the knee joint of a rabbit using autologous cultured osteoblasts. J. Surg. Res..

[125] Hernigou P., Daltro G., Hernigou J. (2018). Hip osteonecrosis: Stem cells for life or behead and arthroplasty?. Int. Orthop..

[126] Chang P.C., Lim L.P., Chong L.Y., Dovban A.S., Chien L.Y., Chung M.C., Lei C., Kao M.J., Chen C.H., Chiang H.C. (2012). Pdgf-simvastatin delivery stimulates osteogenesis in heat-induced osteonecrosis. J. Dent. Res..

[127] Elniel A.R., Giannoudis P.V. (2018). Open fractures of the lower extremity: Current management and clinical outcomes. EFORT Open Rev..

[128] Giannotti S., Trombi L., Bottai V., Ghilardi M., D’Alessandro D., Danti S., Dell’Osso G., Guido G., Petrini M. (2013). Use of autologous human mesenchymal stromal cell/fibrin clot constructs in upper limb non-unions: Long-term assessment. PLoS ONE.

[129] Le Nail L.R., Stanovici J., Fournier J., Splingard M., Domenech J., Rosset P. (2014). Percutaneous grafting with bone marrow autologous concentrate for open tibia fractures: Analysis of forty three cases and literature review. Int. Orthop..

[130] Roffi A., Krishnakumar G.S., Gostynska N., Kon E., Candrian C., Filardo G. (2017). The role of three-dimensional scaffolds in treating long bone defects: Evidence from preclinical and clinical literature—A systematic review. Biomed. Res. Int..

[131] Higuchi T. (1961). Rate of release of medicaments from ointment bases containing drugs in suspension. J. Pharm. Sci..

[132] Peppas N.A. (2013). Historical perspective on advanced drug delivery: How engineering design and mathematical modeling helped the field mature. Adv. Drug Deliv. Rev..

[133] Frenkel D., Smit B. (2002). Understanding Molecular Simulation: From Algorithms to Applications.

[134] Reif M.M., Hunenberger P.H., Oostenbrink C. (2012). New interaction parameters for charged amino acid side chains in the gromos force field. J. Chem. Theory Comput..

[135] Vanommeslaeghe K., Hatcher E., Acharya C., Kundu S., Zhong S., Shim J., Darian E., Guvench O., Lopes P., Vorobyov I. (2010). Charmm general force field: A force field for drug-like molecules compatible with the charmm all-atom additive biological force fields. J. Comput. Chem..

[136] Wang J.M., Wolf R.M., Caldwell J.W., Kollman P.A., Case D.A. (2004). Development and testing of a general amber force field. J. Comput. Chem..

[137] Maier J.A., Martinez C., Kasavajhala K., Wickstrom L., Hauser K.E., Simmerling C. (2015). Ff14sb: Improving the accuracy of protein side chain and backbone parameters from ff99sb. J. Chem. Theory Comput..

[138] Zgarbova M., Otyepka M., Sponer J., Mladek A., Banas P., Cheatham T.E., Jurecka P. (2011). Nucleic acids force field based on reference quantum chemical calculations of glycosidic torsion profiles. J. Chem. Theory Comput..

[139] Dickson C.J., Madej B.D., Skjevik A.A., Betz R.M., Teigen K., Gould I.R., Walker R.C. (2014). Lipid14: The amber lipid force field. J. Chem. Theory Comput..

[140] Kirschner K.N., Yongye A.B., Tschampel S.M., Gonzalez-Outeirino J., Daniels C.R., Foley B.L., Woods R.J. (2008). Glycam06: A generalizable biomolecular force field. Carbohydrates. J. Comput. Chem..

[141] Larsson D.S.D., Liljas L., van der Spoel D. (2012). Virus capsid dissolution studied by microsecond molecular dynamics simulations. PLoS Comput. Biol..

[142] Bernardi R.C., Melo M.C.R., Schulten K. (2015). Enhanced sampling techniques in molecular dynamics simulations of biological systems. Biochim. Biophys. Acta-Gen. Subj..

[143] Wei Z.H., Luijten E. (2015). Systematic coarse-grained modeling of complexation between small interfering rna and polycations. J. Chem. Phys..

[144] Antila H.S., Harkonen M., Sammalkorpi M. (2015). Chemistry specificity of DNA-polycation complex salt response: A simulation study of DNA, polylysine and polyethyleneimine. Phys. Chem. Chem. Phys..

[145] Grasso G., Deriu M.A., Patrulea V., Borchard G., Moller M., Danani A. (2017). Free energy landscape of sirna-polycation complexation: Elucidating the effect of molecular geometry, polymer flexibility, and charge neutralization. PLoS ONE.

[146] Ziebarth J., Wang Y.M. (2009). Molecular dynamics simulations of DNA-polycation complex formation. Biophys. J..

[147] Sun C.B., Tang T., Uludag H. (2012). Molecular dynamics simulations for complexation of DNA with 2 kda pei reveal profound effect of pei architecture on complexation. J. Phys. Chem. B.

[148] Pavan G.M. (2014). Modeling the interaction between dendrimers and nucleic acids: A molecular perspective through hierarchical scales. Chemmedchem.

[149] Pavan G.M., Mintzer M.A., Simanek E.E., Merkel O.M., Kissel T., Danani A. (2010). Computational insights into the interactions between DNA and sirna with “rigid” and “flexible” triazine dendrimers. Biomacromolecules.

[150] Jensen L.B., Pavan G.M., Kasimova M.R., Rutherford S., Danani A., Nielsen H.M., Foged C. (2011). Elucidating the molecular mechanism of pamam-sirna dendriplex self-assembly: Effect of dendrimer charge density. Int. J. Pharm..

[151] Comer J., Schulten K., Chipot C. (2014). Calculation of lipid-bilayer permeabilities using an average force. J. Chem. Theory Comput..

[152] Dickson C.J., Hornak V., Pearlstein R.A., Duca J.S. (2017). Structure-kinetic relationships of passive membrane permeation from multiscale modeling. J. Am. Chem. Soc..

[153] Carpenter T.S., Kirshner D.A., Lau E.Y., Wong S.E., Nilmeier J.P., Lightstone F.C. (2014). A method to predict blood-brain barrier permeability of drug-like compounds using molecular dynamics simulations. Biophys. J..

[154] Bochicchio D., Panizon E., Ferrando R., Monticelli L., Rossi G. (2015). Calculating the free energy of transfer of small solutes into a model lipid membrane: Comparison between metadynamics and umbrella sampling. J. Chem. Phys..

[155] Xiang T.X., Anderson B.D. (2006). Liposomal drug transport: A molecular perspective from molecular dynamics simulations in lipid bilayers. Adv. Drug Deliv. Rev..

[156] Marrink S.J., Berendsen H.J.C. (1996). Permeation process of small molecules across lipid membranes studied by molecular dynamics simulations. J. Phys. Chem..

[157] Marrink S.J., Risselada H.J., Yefimov S., Tieleman D.P., de Vries A.H. (2007). The martini force field: Coarse grained model for biomolecular simulations. J. Phys. Chem. B.

[158] Marrink S.J., Tieleman D.P. (2013). Perspective on the martini model. Chem. Soc. Rev..

[159] Arnarez C., Uusitalo J.J., Masman M.F., Ingolfsson H.I., de Jong D.H., Melo M.N., Periole X., de Vries A.H., Marrink S.J. (2015). Dry martini, a coarse-grained force field for lipid membrane simblations with implicit solvent. J. Chem. Theory Comput..

[160] Walsh T.R. (2017). Pathways to structure-property relationships of peptide-materials interfaces: Challenges in predicting molecular structures. Acc. Chem. Res..

[161] Charchar P., Christofferson A.J., Todorova N., Yarovsky I. (2016). Understanding and designing the gold-bio interface: Insights from simulations. Small.

[162] Nash J.A., Kwansa A.L., Peerless J.S., Kim H.S., Yingling Y.G. (2017). Advances in molecular modeling of nanoparticle nucleic acid interfaces. Bioconjug. Chem..

[163] Tavanti F., Pedone A., Menziani M.C. (2015). Competitive binding of proteins to gold nanoparticles disclosed by molecular dynamics simulations. J. Phys. Chem. C.

[164] Barnard A.S. (2016). Challenges in modelling nanoparticles for drug delivery. J. Phys. Condens. Matter.

[165] Utesch T., Daminelli G., Mroginski M.A. (2011). Molecular dynamics simulations of the adsorption of bone morphogenetic protein-2 on surfaces with medical relevance. Langmuir.

[166] Dong X.L., Qi W., Tao W., Ma L.Y., Fu C.X. (2011). The dynamic behaviours of protein bmp-2 on hydroxyapatite nanoparticles. Mol. Simul..

[167] Siepmann J., Siepmann F. (2008). Mathematical modeling of drug delivery. Int. J. Pharm..

[168] Fredenberg S., Wahlgren M., Reslow M., Axelsson A. (2011). The mechanisms of drug release in poly(lactic-co-glycolic acid)-based drug delivery systems-a review. Int. J. Pharm..

[169] Rossi F., Castiglione F., Salvalaglio M., Ferro M., Moioli M., Mauri E., Masi M., Mele A. (2017). On the parallelism between the mechanisms behind chromatography and drug delivery: The role of interactions with a stationary phase. Phys. Chem. Chem. Phys..

[170] Masaro L., Zhu X.X. (1999). Physical models of diffusion for polymer solutions, gels and solids. Prog. Polym. Sci..

[171] Dobrynin A.V. (2008). Theory and simulations of charged polymers: From solution properties to polymeric nanomaterials. Curr. Opin. Colloid Interface Sci..

[172] Amsden B., Grotheer K., Angl D. (2002). Influence of polymer ionization degree on solute diffusion in polyelectrolyte gels. Macromolecules.

[173] Fatin-Rouge N., Milon A., Buffle J., Goulet R.R., Tessier A. (2003). Diffusion and partitioning of solutes in agarose hydrogels: The relative influence of electrostatic and specific interactions. J. Phys. Chem. B.

[174] Gu W.Y., Yao H., Vega A.L., Flagler D. (2004). Diffusivity of ions in agarose gels and intervertebral disc: Effect of porosity. Ann. Biomed. Eng..

[175] Yan H.J., Casalini T., Hulsart-Billstrom G., Wang S.J., Oommen O.P., Salvalaglio M., Larsson S., Hilborn J., Varghese O.P. (2018). Synthetic design of growth factor sequestering extracellular matrix mimetic hydrogel for promoting in vivo bone formation. Biomaterials.

[176] Siepmann J., Peppas N.A. (2012). Modeling of drug release from delivery systems based on hydroxypropyl methylcellulose (hpmc). Adv. Drug Deliv. Rev..

[177] Siepmann J., Peppas N.A. (2001). Mathematical modeling of controlled drug delivery. Adv. Drug Deliv. Rev..

[178] Lauzon M.A., Bergeron E., Marcos B., Faucheux N. (2012). Bone repair: New developments in growth factor delivery systems and their mathematical modeling. J. Control. Release.

[179] Versypt A.N.F., Pack D.W., Braatz R.D. (2013). Mathematical modeling of drug delivery from autocatalytically degradable plga microspheres—A review. J. Control. Release.

[180] Lao L.L., Peppas N.A., Boey F.Y.C., Venkatraman S.S. (2011). Modeling of drug release from bulk-degrading polymers. Int. J. Pharm..

[181] Casalini T. (2017). Bioresorbability of polymers: Chemistry, mechanisms, and modeling. Bioresorbable Polym. Biomed. Appl..

[182] Alexis F. (2005). Factors affecting the degradation and drug-release mechanism of poly(lactic acid) and poly[(lactic acid)-co-(glycolic acid)]. Polym. Int..

[183] Batycky R.P., Hanes J., Langer R., Edwards D.A. (1997). A theoretical model of erosion and macromolecular drug release from biodegrading microspheres. J. Pharm. Sci..

[184] Nishida H., Yamashita M., Nagashima M., Hattori N., Endo T., Tokiwa Y. (2000). Theoretical prediction of molecular weight on autocatalytic random hydrolysis of aliphatic polyesters. Macromolecules.

[185] Arosio P., Busini V., Perale G., Moscatelli D., Masi M. (2008). A new model of resorbable device degradation and drug release—Part I: Zero order model. Polym. Int..

[186] Perale G., Arosio P., Moscatelli D., Barri V., Muller M., Maccagnan S., Masi M. (2009). A new model of resorbable device degradation and drug release: Transient 1-dimension diffusional model. J. Control. Release.

[187] Casalini T., Rossi F., Lazzari S., Perale G., Masi M. (2014). Mathematical modeling of plga microparticles: From polymer degradation to drug release. Mol. Pharm..

[188] Siepmann J., Faisant N., Benoit J.P. (2002). A new mathematical model quantifying drug release from bioerodible microparticles using monte carlo simulations. Pharm. Res..

[189] Omer A., Suryanarayanan V., Selvaraj C., Singh S.K., Singh P. (2015). Explicit drug re-positioning: Predicting novel drug-target interactions of the shelved molecules with qm/mm based approaches. Adv. Protein. Chem. Struct. Biol..

[190] Ganesan A., Coote M.L., Barakat K. (2017). Molecular dynamics-driven drug discovery: Leaping forward with confidence. Drug Dis. Today.

[191] Kitchen D.B., Decornez H., Furr J.R., Bajorath J. (2004). Docking and scoring in virtual screening for drug discovery: Methods and applications. Nat. Rev. Drug Dis..

[192] Aqvist J., Medina C., Samuelsson J.E. (1994). New method for predicting binding-affinity in computer-aided drug design. Protein Eng..

[193] Genheden S., Ryde U. (2015). The mm/pbsa and mm/gbsa methods to estimate ligand-binding affinities. Expert Opin. Drug Dis..

[194] Chodera J.D., Mobley D.L., Shirts M.R., Dixon R.W., Branson K., Pande V.S. (2011). Alchemical free energy methods for drug discovery: Progress and challenges. Curr. Opin. Struct. Biol..

[195] Wang C.H., Nguyen P.H., Pham K., Huynh D., Le T.B.N., Wang H.L., Ren P.Y., Luo R. (2016). Calculating protein-ligand binding affinities with mmpbsa: Method and error analysis. J. Comput. Chem..

[196] Siepmann J., Siepmann F. (2012). Modeling of diffusion controlled drug delivery. J. Control. Release.

[197] Korsmeyer R.W., Lustig S.R., Peppas N.A. (1986). Solute and penetrant diffusion in swellable polymers. 1. Mathematical-modeling. J. Polym. Sci. Part B.

[198] Korsmeyer R.W., Vonmeerwall E., Peppas N.A. (1986). Solute and penetrant diffusion in swellable polymers. 2. Verification of theoretical-models. J. Polym. Sci. Part B.

[199] Lee P.I. (2011). Modeling of drug release from matrix systems involving moving boundaries: Approximate analytical solutions. Int. J. Pharm..

[200] Sackett C.K., Narasimhan B. (2011). Mathematical modeling of polymer erosion: Consequences for drug delivery. Int. J. Pharm..

[201] Antheunis H., van der Meer J.C., de Geus M., Heise A., Koning C.E. (2010). Autocatalytic equation describing the change in molecular weight during hydrolytic degradation of aliphatic polyesters. Biomacromolecules.

[202] Rothstein S.N., Federspiel W.J., Little S.R. (2009). A unified mathematical model for the prediction of controlled release from surface and bulk eroding polymer matrices. Biomaterials.

[203] Ramkrishna D. (2000). Population Balances: Theory and Applications to Particulate Systems in Engineering.

